# TCF21/POD-1, a Transcritional Regulator of SF-1/NR5A1, as a Potential Prognosis Marker in Adult and Pediatric Adrenocortical Tumors

**DOI:** 10.3389/fendo.2018.00038

**Published:** 2018-02-22

**Authors:** Barbara dos Santos Passaia, Matheus Henrique Dias, Jean Lucas Kremer, Sonir Roberto Rauber Antonini, Madson Queiroz de Almeida, Maria Candida Barisson Villares Fragoso, Claudimara Ferini Pacicco Lotfi

**Affiliations:** ^1^Department of Anatomy, Institute of Biomedical Science, University of São Paulo, São Paulo, Brazil; ^2^Special Laboratory of Applied Toxicology (LETA), Butantan Institute, São Paulo, Brazil; ^3^Department of Pediatrics and Puericulture, School of Medicine of Ribeirão Preto, University of São Paulo, Ribeirão Preto, Brazil; ^4^Adrenal Unit, Hormone and Molecular Genetic Laboratory/LIM42, Hospital of Clinics, School of Medicine, University of São Paulo, São Paulo, Brazil

**Keywords:** adrenocortical tumors, adult and pediatric tumors, transcription factor 21, podocyte-expressed 1, BUB1B, PTEN-induced putative kinase 1, nuclear receptor subfamily 5 group A member 1, CRISPR/dCas9

## Abstract

With recent progress in understanding the pathogenesis of adrenocortical tumors (ACTs), identification of molecular markers to predict their prognosis has become possible. Transcription factor 21 (TCF21)/podocyte-expressed 1 (POD1) is a transcriptional regulatory protein expressed in mesenchymal cells at sites of epithelial–mesenchymal transition during the development of different systems. Adult carcinomas express less *TCF21* than adenomas, in addition, the KEGG pathway analysis has shown that *BUB1B*, among others genes, is negatively correlated with *TCF21* expression. The difference between *BUB1B* and PTEN-induced putative kinase 1 (*PINK1*) expression has been described previously to be associated with survival in adult but not in pediatric carcinomas. Here, we analyzed the gene expression of *TCF21, BUB1B, PINK1*, and *NR5A1* in adult and pediatric ACTs. We found a negative correlation between the relative expression levels of *TCF21* and *BUB1B* in adult ACTs, but the relative expression levels of *TCF21, BUB1B, PINK1*, and *NR5A1* were similar in childhood ACTs. In addition, we propose using the subtracted expression levels of the *TCF21/POD-1* genes as a predictor of overall survival (OS) in adult carcinomas and *TCF21-NR5A1* as a predictor of malignancy for pediatric tumors in patients aged <5 years. These results require further validation in different cohorts of both adult and pediatric samples. Finally, we observed that the OS for patients aged <5 years was markedly favorable compared with that for patients >5 years as well as adult patients with carcinoma. In summary, we propose *TCF21/POD-1* as a new prognostic marker in adult and pediatric ACTs.

## Introduction

The molecular pathogenesis of adrenocortical tumors (ACTs) remains poorly understood despite recent advances provided by comprehensive clinical and molecular investigations (1, 2). Some advances are related to the development of different transcriptomes during the last decade [for a review, see Ref. (3)]. Among these studies, a microarray analysis (4) showed that 91 genes are differentially expressed between adrenocortical carcinomas (ACCs) and adrenocortical adenomas (ACAs) of adult patients, including the *TCF21* gene, which was two times lower in ACCs than in ACAs or in normal adrenal cortex samples. Transcription factor 21 (TCF21)/podocyte-expressed 1 (POD1) also known as capsulin/epicardin is a bHLH transcriptional regulatory protein expressed at sites of epithelial–mesenchymal interactions in the developing urogenital, cardiovascular, respiratory, and gastrointestinal systems (5–9). TCF21 directly regulates the expression of steroidogenic factor 1 (*NR5A1*/*SF1*) in human ACT cells by binding to the E-box sequence in the *NR5A1’s* promoter region (10). Also, in this study, we showed that the viability of ACC cells transfected with *TCF21* was not affected. However, StAR expression was downregulated following transfection with *TCF21*, in accordance with a decrease in SF1-mediated StAR transcription. In addition, KEGG analysis showed a significant enrichment in cell cycle regulation pathways involving genes whose expression was negatively correlated with *TCF21* expression in ACCs, such as *CDK1* and *BUB1B* (10). Budding uninhibited by benzimidazoles 1 homolog beta (*BUB1B*) encodes a kinase with important functions in the mitotic checkpoint (11–13). In a study using microarrays to identify genes that discriminated ACC and ACA, they identified two clusters of ACC with different outcomes (14). de Reyniès and colleagues identified that the difference between the expression values of *DLGAP5* (disks large associated protein 7) and *PINK1* (PTEN induced putative kinase 1), and the difference between the expression of values of *BUB1B* and *PINK* were, respectively, predictors of malignancy, and overall survival. Indeed, Δ*C*_T_*BUB1B −* Δ*C*_T_*PINK1* was considered a prognostic factor in ACCs in two different cohorts (14, 15).

PTEN-induced putative kinase 1 (PINK1) is a key mediator of mitochondria quality control induced by the tumor suppressor gene PTEN (16), whose expression levels decline in more aggressive ACCs and in ovarian cancer (17).

For pediatric patients, there are limited data to define prognostic molecular markers that distinguish benign from malignant ACTs, despite attempts of histological criteria and molecular classifications (18, 19). In fact, the only study using transcriptome profiling analysis of pediatric ACT did not discriminate ACAs and ACCs using unsupervised clustering (20). Therefore, due to clinical, histological, and molecular heterogeneity in malignant ACTs, there is a need to validate and correlate the driver genes with possible prognostic value for both adult and pediatric ACTs.

In this study, we aimed to analyze the expression of *TCF21* and genes that may have *TCF21*-related expression, such as *BUB1B, PINK1*, and *NR5A1* in adult and pediatric ACTs. Through the analysis of *TCF21, BUB1B, PINK1*, and *NR5A1* gene expression, we tested the value of this analysis to predict the OS of adult ACC and to distinguish between benign and malignant pediatric ACTs. Therefore, the overall aim of this study was to verify if *TCF21* has a diagnostic and prognostic role in adult and pediatric tumors.

## Materials and Methods

### Patients

This study was approved by the Ethics Committees of Hospital das Clinicas, Institute of Biomedical Sciences (#822/2016) and Department of Pediatrics and School of Medicine of Ribeirão Preto (#7534/2010), São Paulo, Brazil. Written informed consent was obtained from all the patients or from their parents.

The clinical and histological features of patients with ACTs are summarized in Table 1. Further details of clinical characteristics and molecular data are shown in Tables S1 and S2 in Supplementary Material.

**Table 1:** Clinical characteristic of patients.

Adults	Characteristics	*n* = 78	Adrenocortical adenoma (ACA) (*n* = 44)	Adrenocortical carcinoma (ACC) (*n* = 34)
	Mean age (years)		40.59 ± 13.8	41.68 ± 16.45
	Sex	Female	36	23
		Male	8	11
	Weiss score^b^	≤3	42	7
		>3	1	27
	ENSAT stage^c^	I/II	1	11
		III/IV	0	10
	Metastasis^b^	Yes	0	16
		No	32	14
	Cancer-related death (CRD)^a^		1	15
	Median OS (mo)			41
	Follow-up (mo)		59.4 ± 62.41	52.2 ± 69.94

**Pediatric**	**<5 years**	***n* = 35**	**ACA (*n* = 27)**	**ACC (*n* = 8)**

	Mean age (years)		1.84 ± 0.76	2.44 ± 0.87
	Sex	Female	19	4
		Male	8	4
	Weiss Score^b^	≤3	9	0
		>3	17	8
	Metastasis^d^	Yes	0	7
		No	26	1
	CRD		0	5
	Median OS (mo)			197.7
	Mean follow-up (months)		100.2 ± 61.9	57.67 ± 65.05

**Pediatric**	**>5 years**	***n* = 15**	**ACA (*n* = 5)**	**ACC (*n* = 10)**

	Mean age (years)		10.4 ± 4.03	13.44 ± 3.98
	Sex	Female	4	8
		Male	1	2
	Weiss score	≤3	5	0
		>3	0	10
	Metastasis	Yes	0	7
		No	5	3
	CRD		0	7
	Median OS (mo)			23.3
	Mean follow-up (months)		96.5 ± 61.14	30.21 ± 34.7

*^a^11 cases not informed*.

*^b^1 case not informed*.

*^c^55 cases not informed*.

*^d^16 cases not informed*.

*OS, overall survival*.

Samples of ACTs were obtained from 128 patients, 78 adult patients (range: 18–83 years), 35 pediatric patients aged <5 years (range: 0.43–3.8 years), and 15 pediatric patients aged >5 years (range: 5.5–17.7 years). The pediatric patients were separated into two groups because the fetal zone of the human adrenal cortex undergoes involution after birth, with the glomerulosa and fasciculata zones achieving complete differentiation approximately 4 years of age (21, 22), while the reticularis zone is formed from 6 to 9 years of age (23, 24). Patients were evaluated at Hospital das Clinicas, by the School of Medicine from University of São Paulo and at the Department of Pediatrics from the School of Medicine of Ribeirao Preto, São Paulo, Brazil, between 1981 and 2014.

The mean follow-up periods were 56.2 ± 65.9, 90.5 ± 65.1, and 52.3 ± 55.0 months for adult patients, pediatric patients aged <5 years, and pediatric patients aged >5 years, respectively. The final diagnosis of the patients was determined according to the histopathological characteristics, clinical manifestation, and biological behavior of the tumor, as proposed by Wieneke et al. (18), and it was used to classify the tumors as adult and pediatric adenomas or carcinomas in this study. As shown in Table 1, the Weiss score for adult ACTs was different from the correct diagnosis based on the final diagnosis. Accordingly, in our study, we used the final diagnosis (44 ACA; 34 ACC) instead of the Weiss score (49 ACA; 28 ACC) to classify adenomas and carcinomas for adult tumors. For the pediatric group, the Weiss criteria are not useful to discriminate the histopathological diagnosis because pediatric tumors present a favorable outcome even with a Weiss score ≥3 (18). Among pediatric patients up to 5 years of age, the final diagnosis was 27 ACAs and 8 ACCs; however, among pediatric patients from 5 to 18 years of age, 5 ACAs and 10 ACCs were diagnosed (Table 1).

### Cell Cultures

Human ACC cell lines NCI-H295R (25) and SW-13 (26) and human embryonic kidney cell line HEK-293 (27) were obtained from ATCC (The ATCC Cell Biology Collection). NCI-H295R, SW-13, and HEK-293 were cultured, respectively, in RPMI medium with 2% fetal bovine serum (FBS) and 1% insulin-transferrin-selenium, L-15 medium with 10% FBS, and DMEM medium with 10% FBS (Gibco, Grand Island, NY, USA) at 37°C in a 95% air-5% CO_2_, in fully humidified environment. The culture used was authenticated by STR DNA profiling analysis.

### Quantitative Real-time PCR

Total RNA was extracted from previously frozen tumor fragments (stored in liquid nitrogen) using Trizol (Invitrogen, Carlsbad, CA, USA) and an automatic homogenizer (model 985370, Biospec Products, Bartlesville, OK, USA). The RNA integrity and concentration were evaluated by agarose gel electrophoresis (2%) and spectrometry (NanoDrop 2000c, Thermo Fisher Scientific, Waltham, MA, USA). cDNA was generated from 1 µg of RNA using the SuperScript III First-Strand Synthesis Supermix kit (Invitrogen). Quantitative real-time PCR was performed using the 7500 Real Time PCR System Sequencer (Applied Biosystems, Foster City, CA, USA) and the TaqMan gene expression assay for gene quantification according to the manufacturer’s instructions (Applied Biosystems, Foster City, CA, USA). The assays IDs were as follows: human β-glucuronidase, glucuronidase beta (GUSB) (Hs00939627_m1 ID), beta-actin (ACTB) (Hs99999903_m1 ID), TCF21 (Hs00162646_m1 ID), nuclear receptor subfamily 5 group A member 1 (NR5A1) (ID Hs00610436_m1), BUB1B (ID Hs01084828_m1), and PINK1 (ID Hs00260868_m1). A cycle threshold (CT) value was selected in the linear range of amplification for each sample in triplicate and was normalized to the GUSB and ACTB expression levels. The relative expression levels were calculated using the 2^−ΔΔCt^ method (28), where ΔΔCt is the difference between the selected ΔCt value of a given sample and the ΔCt for a pool of commercial normal adrenals (BioChain, USA). For the pediatric groups, the ΔCt mean of eight pediatric normal adrenal samples were used as normalizer. These samples were collected from patients up to 5 years of age undergoing nephrectomy due to kidney cancer and were kindly provided by Dr. Sonir R. R. Antonini from the Department of Pediatrics of FMRP-USP.

A mean expression value of 1.0 was attributed to the target genes in the pool of normal adrenals. A relative increase in the expression levels was determined for each tumor sample. The subtraction gene level expression was calculated using the formula ΔCt target gene 1 − ΔCt target gene 2, as described by de Reyniès et al. (14).

### Transfection Assay

NCI-H295R cells were transiently transfected with pcMVMycPod1, which was kindly provided by Dr. Masataka Nakamura (Tokyo Medical University, Japan), as described earlier by Funato et al. (29). Next, 1.1 × 10^6^ cells were plated and transfected with 4 µg of plasmid DNA and 12 µl of Turbofect (Thermo Fisher Scientific, Waltham, MA, USA) for 5 h. After 24 h of transfection, total RNA was extracted with Trizol (Invitrogen). Three independent experiments were performed. A high (71.4 ± 2.8%) efficiency of transfection was verified using the pmaxGFP vector (Amaxa Biosystems, Gaithersburg, MD, USA), and the cells were analyzed in a fluorescence-inverted microscope (data not shown).

### Transduction Assay for CRISPR/dCas9 Activation System

The lentiviruses were produced in HEK-293FT cells using plasmids sgRNA (MS2) (#61427; Addgene, Cambridge, MA, USA), dCas9-VP64 (#61425; Addgene, Cambridge, MA, USA), or MS2-P65-HSF1 (#61426; Addgene, Cambridge, MA, USA). The sgRNA sramble (SCR) was constructed with the sequence GCACTACCAGAGCTAACTCA and the sgRNA T2 with the sequence ACATTACAAGTTGCAAATCA, according to protocol established by Konermann et al. (30).

Transduction and cell selection were performed serially: dCas9-VP64 was selected with blasticidin; MS2-P65-HSF1 was selected with hygromycin; and sgRNA-SCR or sgRNA-T2 was selected with zeocin. The concentration of antibiotics used was determined through a dose–response curve. The cells were plated to reach 50% of confluency 48 h before transduction and maintained for 24 h with a solution (1:1) of viral supernatant in culture medium, followed by of antibiotic selection until control cells died.

### Statistical Analysis

As described in the Section “Materials and Methods” (patients), the groups of adenomas and carcinomas were discriminated considering the final diagnosis. The data are presented as medians for the Mann–Whitney test or mean ± SD for Student’s *t* test, as indicated in the figure legends. The ROC curve analysis method was used to test the combined gene expression as molecular predictors. The cut-off obtained was selected considering the best sensitivity and specificity. Due to the limitation to obtain tumor samples, cut-off points were obtained and tested in the same cohort. The log-rank test was used to demonstrate the applicability of the cut-off in the groups analyzed. The event considered in the OS curve was cancer-related death. The critical value for significance of *P* < 0.05 was used throughout the study.

## Results

### Relative Expression Levels of *TCF21, BUB1B, PINK1*, and *NR5A1* in Samples of Adult and Pediatric ACTs

**Figure 1:**
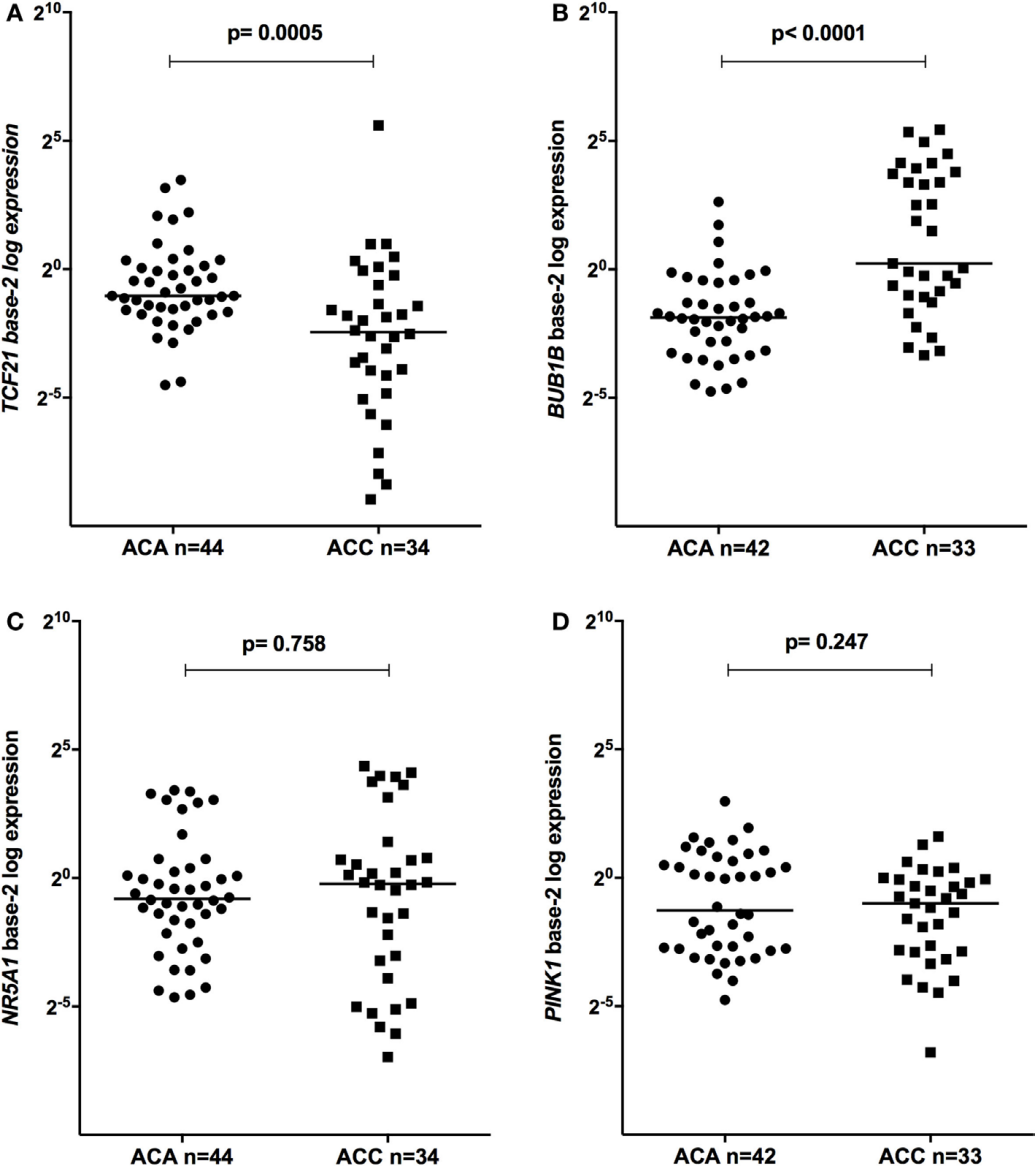
Relative expression of **(A)** transcription factor 21 (TCF21) in 44 adult adrenocortical adenomas (ACAs) and 34 carcinomas (ACCs) [median ACAs = 0.49, median ACCs = 0.18, 95% confidence interval (CI) = −0.46 to −0.13]; **(B)** *BUB1B* in 42 ACAs and 33 ACCs (median ACAs = 0.27, median ACCs = 1.17, 95% CI = 0.43 to 5.70); **(C)** *NR5A1* in 44 adult ACAs and 34 ACCs (median ACAs = 0.86, median ACCs = 0.57, 95% CI = −0.25 to 0.65); and **(D)** *PINK1* in 42 ACAs and 33 ACCs (median ACAs = 0.50, median ACCs = 0.42, 95% CI = −0.53 to 0.11) by quantitative real-time PCR. The *Y*-axis shows the fold increase in gene expression relative to the mean expression of a pool of normal adrenals. The Mann–Whitney test was used to assess statistical significance (*P* < 0.05).

**Figure 2:**
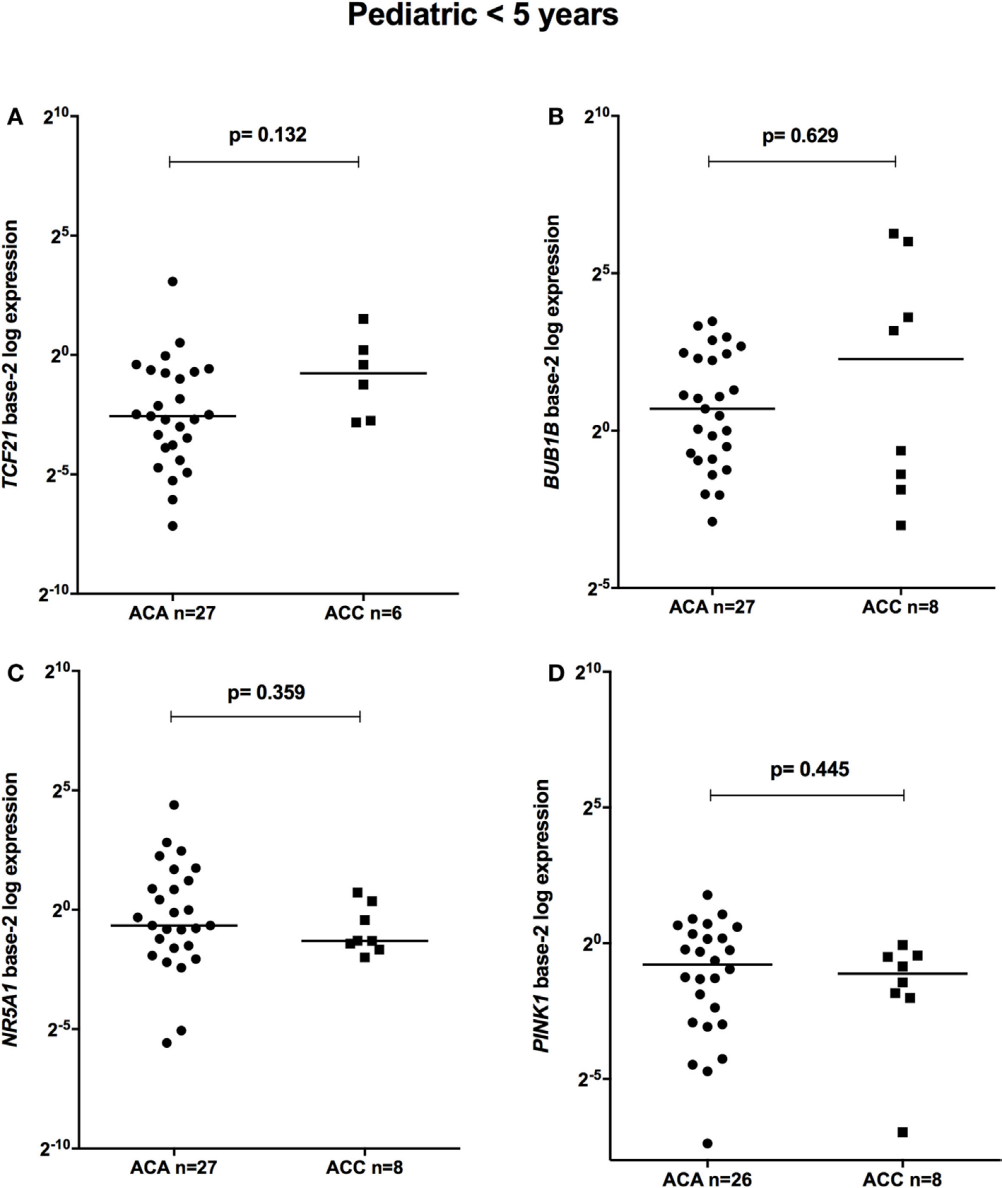
Relative expression of **(A)** *TCF21* in 27 pediatric (<5 years) adrenocortical adenomas (ACAs) and 6 carcinomas (ACCs) [median ACA = 0.17, median ACC = 0.59, 95% confidence interval (CI) = −0.04 to 1.00]; **(B)** *BUB1B* in 27 ACAs and 8 ACCs (median ACAs = 1.62, median ACCs = 4.85, 95% CI = −1.12 to 11.8); **(C)** *NR5A1* in 27 ACAs and 8 ACCs (median ACAs = 0.64, median ACCs = 0.41, 95% CI = −1.48 to 0.19); and **(D)** *PINK1* in 26 ACAs and 8 ACCs (median ACAs = 0.58, median ACCs = 0.46, 95% CI = −0.81 to 0.24) by quantitative real-time PCR. The *Y*-axis shows the fold increase in gene expression relative to the mean expression of a pool of eight pediatric normal adrenals. The Mann–Whitney test was used to assess statistical significance (*P* < 0.05).

**Figure 3:**
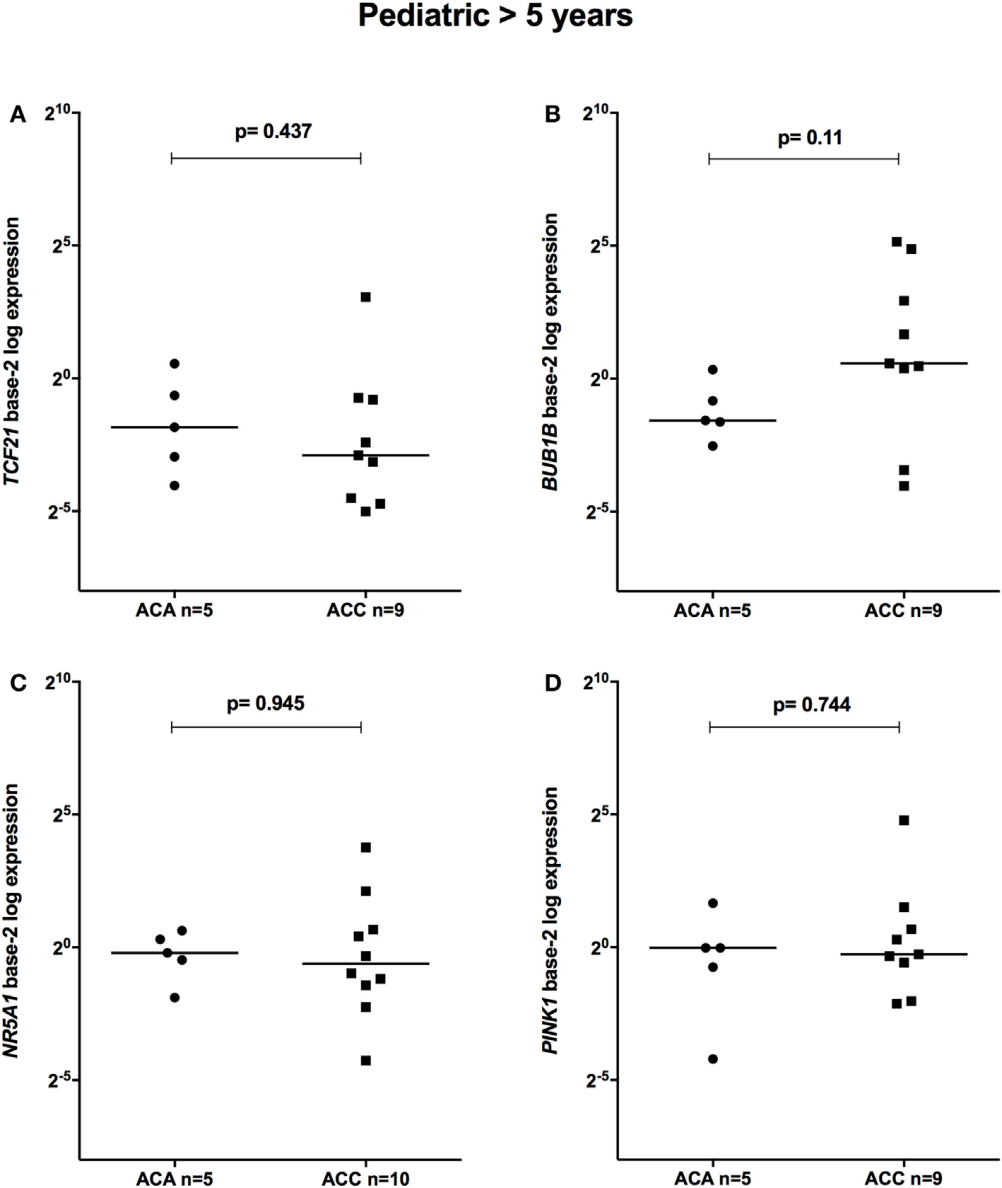
Relative expression of **(A)** *TCF21* in 5 pediatric (>5 years) adrenocortical adenomas (ACAs) and 9 carcinomas (ACCs) [median ACAs = 0.28, median ACCs = 0.13, 95% confidence interval (CI) = −0.87 to 0.47]; **(B)** *BUB1B* in 5 ACAs and 9 ACCs (median ACAs = 0.34, median ACCs = 1.49, 95% CI = −0.24 to 29.07); **(C)** *NR5A1* in 5 ACAs and 10 ACCs (median ACAs = 0.87, median ACCs = 0.65, 95% CI = −0.81 to 3.10); and **(D)** *PINK1* in 5 ACAs and 9 ACCs (median ACAs = 0.98, median ACCs = 0.83, 95% CI = −0.75 to 1.86) by quantitative real-time PCR. The *Y*-axis shows the fold increase in gene expression relative to the mean expression of a pool of eight pediatric normal adrenals. The Mann–Whitney test was used to assess statistical significance (*P* < 0.05).

The relative expression of *TCF21* transcripts was higher in adult ACA than in ACC (0.49 vs 0.18; *P* = 0.0005) (Figure 1A), in line with previous studies (4, 10). By contrast, the relative expression of *BUB1B* transcripts was higher in ACC than in ACA (1.17 vs 0.27; *P* < 0.0001) (Figure 1B), suggesting a negative correlation between *TCF21* and *BUB1B*, as previously proposed (10). The relative expression of *NR5A1* and *PINK1* (Figures 1C,D) was similar in adult ACC and ACA (0.57 vs 0.86; *P* = 0.758; 0.42 vs 0.5; *P* = 0.247, respectively). In addition, the relative expression levels of *TCF21, BUB1B, PINK1*, and *NR5A1* were similar in childhood ACTs, regardless of the group studied (Figures 2 and 3).

**Figure 4:**
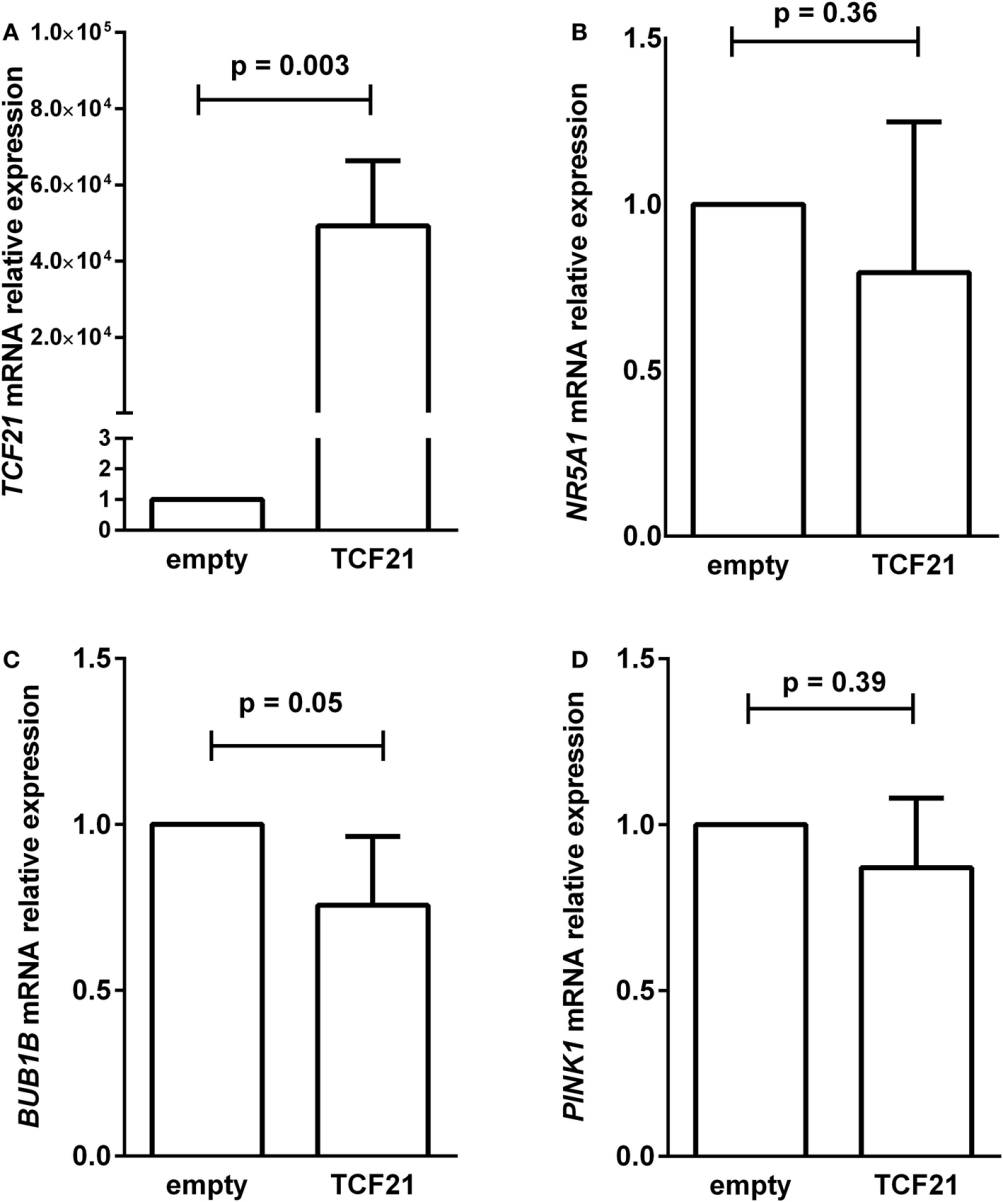
RT-qPCR analysis to determine the relative gene expression of **(A)** *TCF21* [95% confidence interval (CI) = 27,888 to 70,495], **(B)** *NR5A1* (CI = −0.77 to 0.36), **(C)** *BUB1B* (CI = −0.50 to 0.01), and **(D)** *PINK1* (CI = −0.65 to 0.39) in H295R cells transiently transfected with an empty vector pCMVMyc (empty) vs pCMVMycPod1 (*TCF21*). Statistical significance was assessed by paired *T* test on three to five pairs.

To test whether the induction of *TCF21* expression affected *BUB1B* or *PINK1* expression in the NCI-H295R ACC cell line, we performed RT-qPCR of *BUB1B* or *PINK1* in cells transiently transfected with the expression vector pCMVMycPOD1. NCI-H295R cells transfected with pcCMVMycPOD1 (Figure 4A) did not significantly affect *NR5A1* expression (Figure 4B), in contrast to that observed previously (10), probably due to the experimental variation obtained. In addition, pcCMVMycPOD1 transfection showed a tendency of reduction in *BUB1B* expression (Figure 4C), but did not significantly affect *PINK1* expression (Figure 4D) compared with the control levels. The same test with different biological approach was performed in HEK-293 and SW-13 cell lines transduced with CRISPR/Cas9/TCF21 activation system, and TCF21 showed not reduction of BUB1B expression (Figure 10).

### Molecular Predictors of Malignancy in Adult ACTs

**Figure 5:**
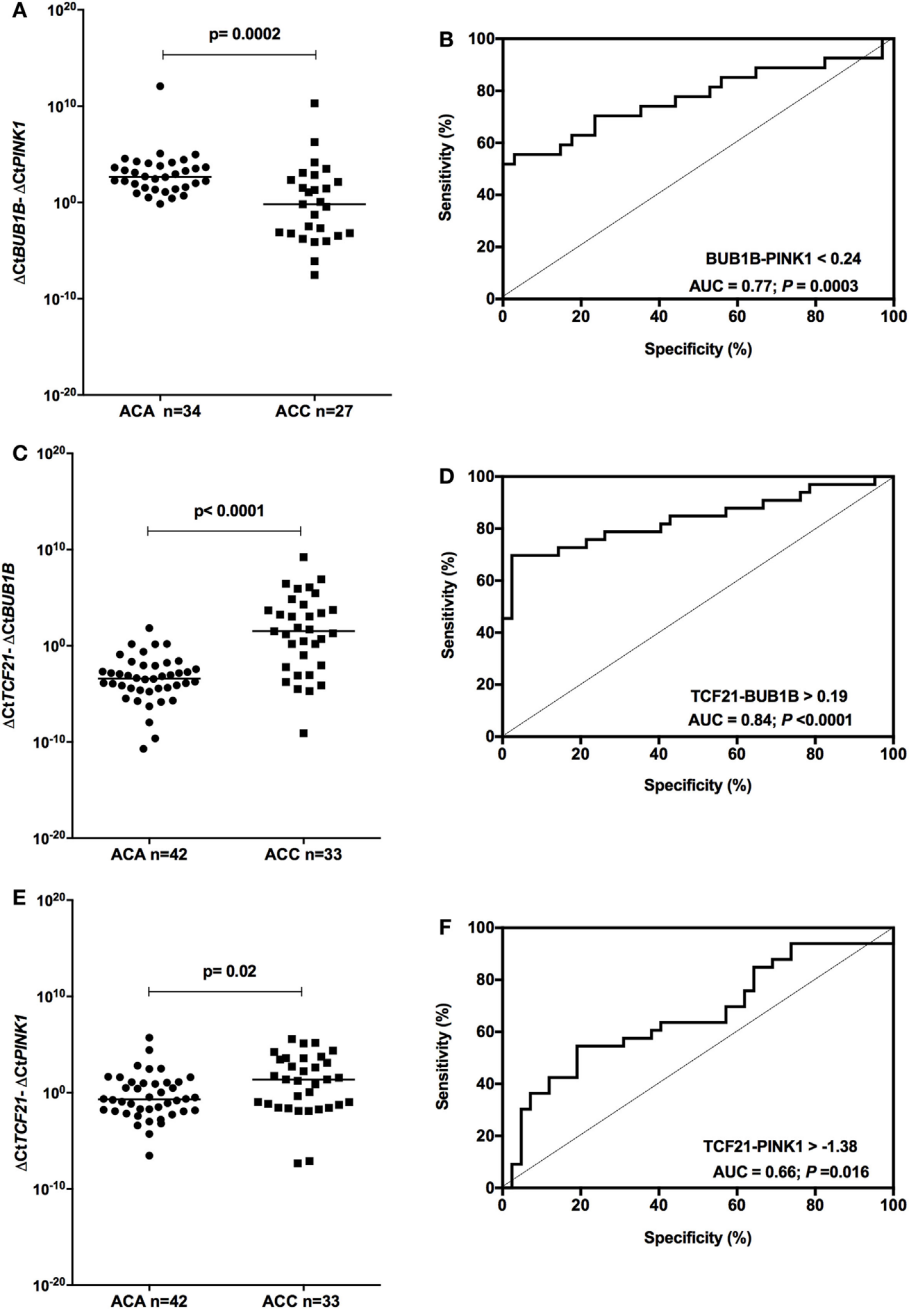
Analysis of the subtraction gene expression of adult adenomas (ACAs) and carcinomas (ACCs) to determine the prognosis. **(A)** Δ*CtBUB1B* and Δ*CtPINK1* expression [median = −3.137; 95% confidence interval (CI): −4.932 to −1.397; *P* = 0.0003] and **(B)** ROC curve test for *BUB1B-PINK1*; **(C)** Δ*CtTCF21* and Δ*CtBUB1B* expression (median = 4.985; 95% CI: 3.376 to 6.502; *P* < 0.0001) and **(D)** ROC curve test for *TCF21-BUB1B*; **(E)** Δ*CtTCF21* and Δ*CtPINK1* expression (median = 1.538; 95% CI: 0.269 to 2.835; *P* = 0.016) and **(F)** ROC curve test for *TCF21-PINK1*.

We tested whether the *ΔCtBUB1B* − *ΔCtPINK1* could discriminate between adenomas and carcinomas in our cohort. After removing from the analysis the patients whose data overlapped with the previous study of our group (15), we found significant differences (*P* = 0.0002; Mann–Whitney test) in the expression level of *BUB1B-PINK1* (Figure 5A) between patients whose final diagnosis was adenoma (median = 2.67; *n* = 34) and those whose final diagnosis was carcinoma (median = −0.176; *n* = 27). To select the cut-off for *BUB1B-PINK1* in our cohort, we applied the ROC curve analysis method (Figure 5B). The area under the curve (AUC) was 0.77 [95% confidence interval (CI): 0.65 to 0.90; *P* = 0.0003]. The cut-off determined was <0.24, with 55.6% of sensitivity and 97.1% of specificity.

Applying the same reasoning, we tested whether subtracting the expression level of *TCF21* and *BUB1B* (*ΔCtTCF21* − *ΔCtBUB1B*) could also discriminate between benign and malignant adult ACTs in the total cohort of adult patients. We found significant differences (*P* < 0.001; Mann–Whitney test) in the expression level of *TCF21-BUB1B* (Figure 5C) between adenomas (median = −3.41; *n* = 42) and carcinomas (median = 1.52; *n* = 33). The AUC obtained from the ROC curve test was 0.84 (95% CI: 0.74 to 0.94; *P* < 0.0001; Figure 5D), and the cut-off determined was >0.19, with 69.7% of sensitivity and 97.6% of specificity. The analysis of *ΔCtTCF21* − *ΔCtPINK1* (Figure 5E) showed significant differences (*P* = 0.02; Mann–Whitney test) between ACA (median = −0.71; *n* = 42) and ACC (median = 1.36; *n* = 33). The AUC obtained from the ROC curve test was 0.66 (95% CI: 0.54 to 0.79; *P* = 0.016; Figure 5F), and the cut-off determined was >−1.38 with 75.8% of sensitivity and 38.1% of specificity.

### *TCF21-BUB1B* As a Predictor of OS in Adult Carcinomas

**Figure 6:**
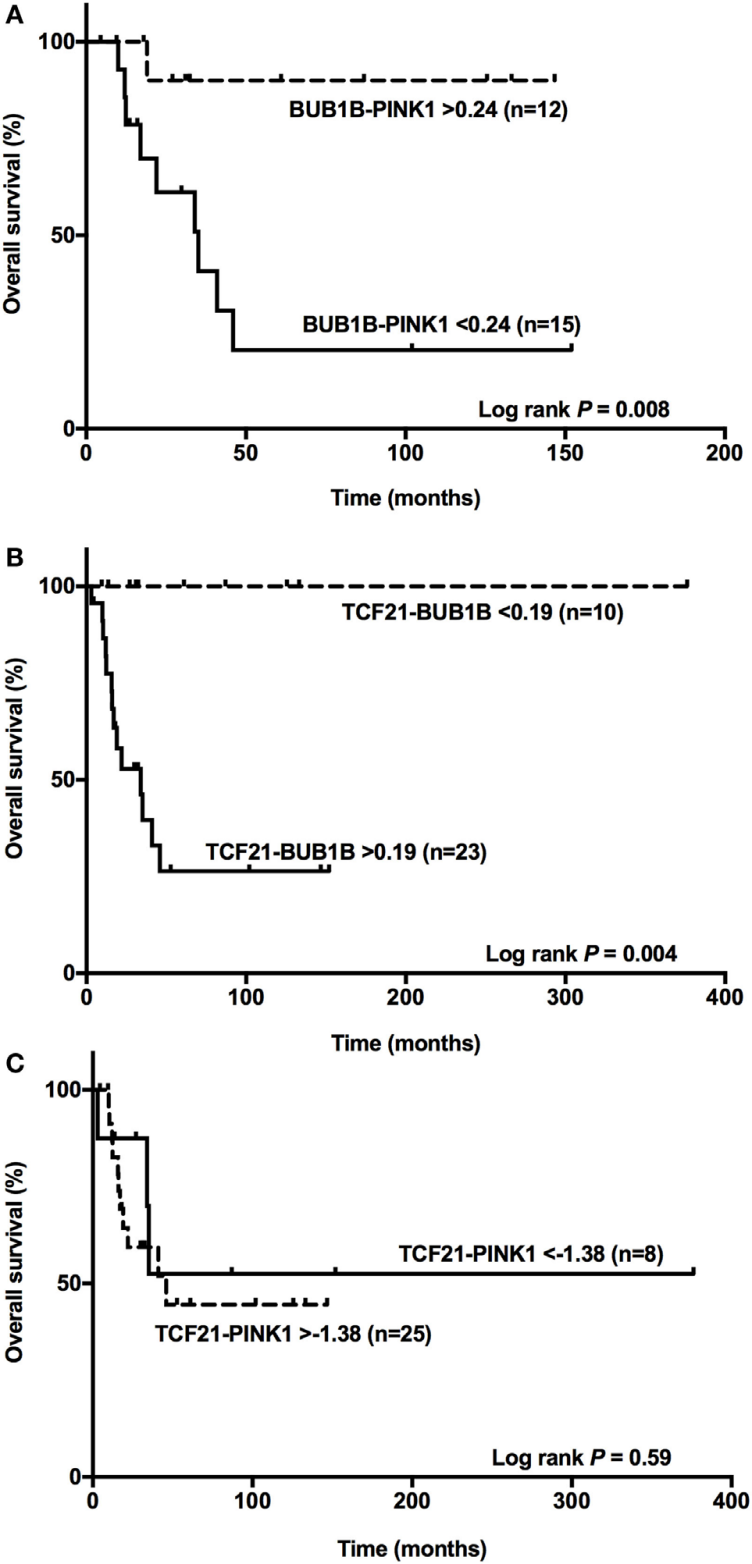
*BUB1B-PINK1, TCF21-BUB1B*, and *TCF21-PINK1* as predictors of overall survival. **(A)** The difference between *BUB1B* and *PINK1* <0.24 significantly (*P* = 0.008) discriminates 15 adult patients with a poor prognosis among 27 adrenocortical carcinomas (ACCs); **(B)** the difference between *TCF21* and *BUB1B* >0.19 significantly (*P* = 0.004) discriminates 23 adult patients with a poor prognosis among 33 ACCs; and **(C)** the difference between *TCF21* and *PINK1* >−1.38 does not discriminate significantly (*P* = 0.59) among 33 ACC patients.

Among the ACCs, subtraction of the expression level of *BUB1B* and *PINK1* (*ΔCtBUB1B* − *ΔCtPINK1*) was a good predictor of OS (14, 15). Applying the cutoff value <0.24 obtained for *BUB1B-PINK1* to 27 adult patients with a malignant final diagnosis (Figure 6A), we found two groups with different survival times (log-rank test *P* = 0.008). Accordingly, we applied the cut-off value >0.19 obtained for *TCF21-BUB1B* to 33 adult patients with a malignant final diagnosis (Figure 6B), and we could discriminate two groups with distinct survival times (log-rank test *P* = 0.004). The cutoff value >−1.38 obtained for *TCF21-PINK1* was not associated with a statistically significant difference in OS (Figure 6C).

### *TCF21-NR5A1* As a Molecular Predictor of Malignancy in Pediatric ACTs

**Figure 7:**
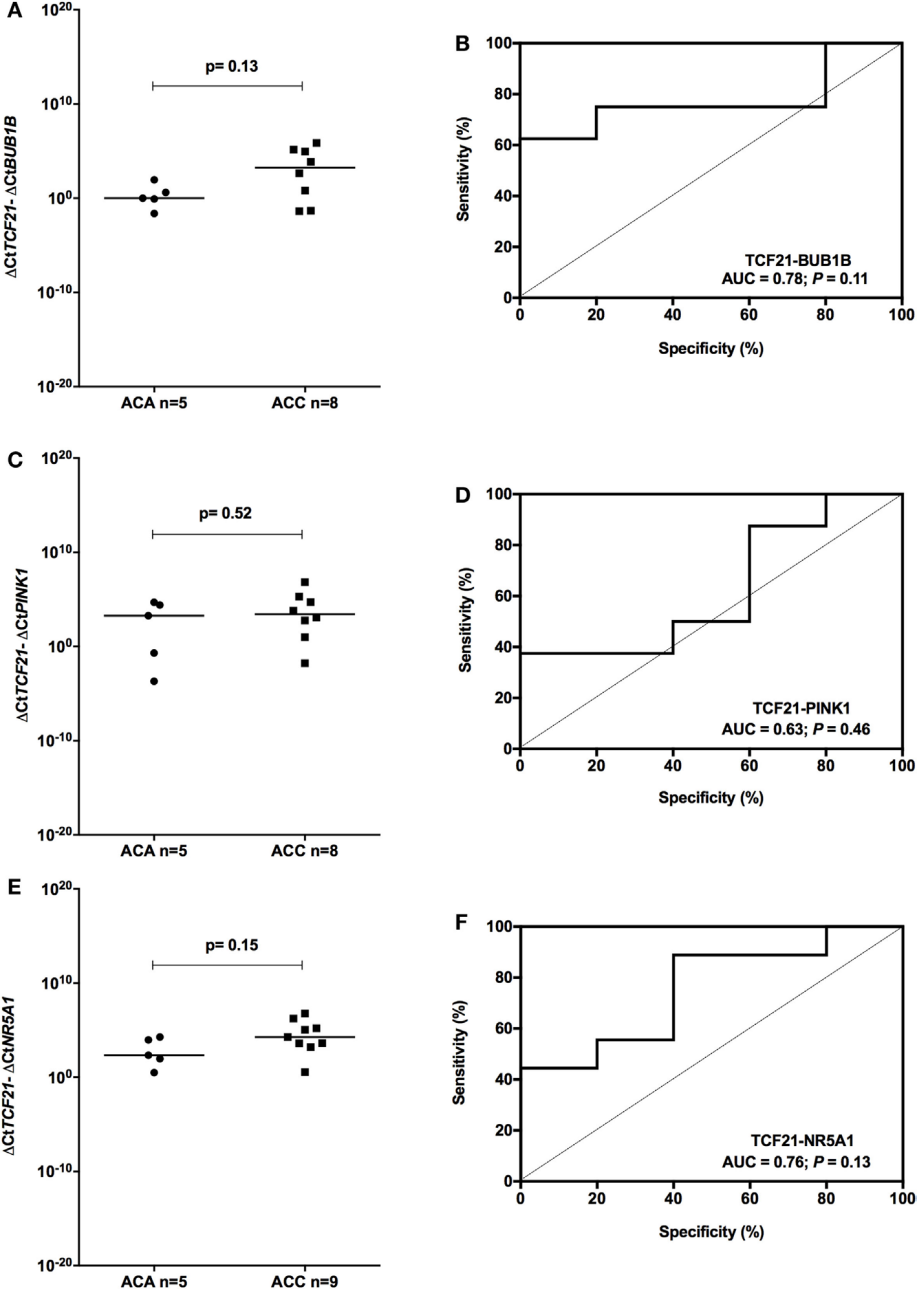
Analysis of the subtraction gene expression of pediatric patients aged >5 years with adenomas (ACAs) and carcinomas (ACCs) to determine the prognosis. **(A)** Δ*CtTCF21* and Δ*CtBUB1B* expression [median = 2.871; 95% confidence interval (CI): −1.284 to 5.267; *P* = 0.13] and **(B)** ROC curve test for *TCF21-BUB1B*; **(C)** Δ*CtTCF21* and Δ*CtPINK1* expression (median = 1.160; 95% CI: −1.928 to 6.446; *P* = 0.52) and **(D)** ROC curve test for *TCF21-PINK1*; **(E)** Δ*CtTCF21* and Δ*CtNR5A1* expression (median = 1.650; 95% CI: −0.650 to 3.905; *P* = 0.15) and **(F)** ROC curve test for *TCF21-NR5A1*.

**Figure 8:**
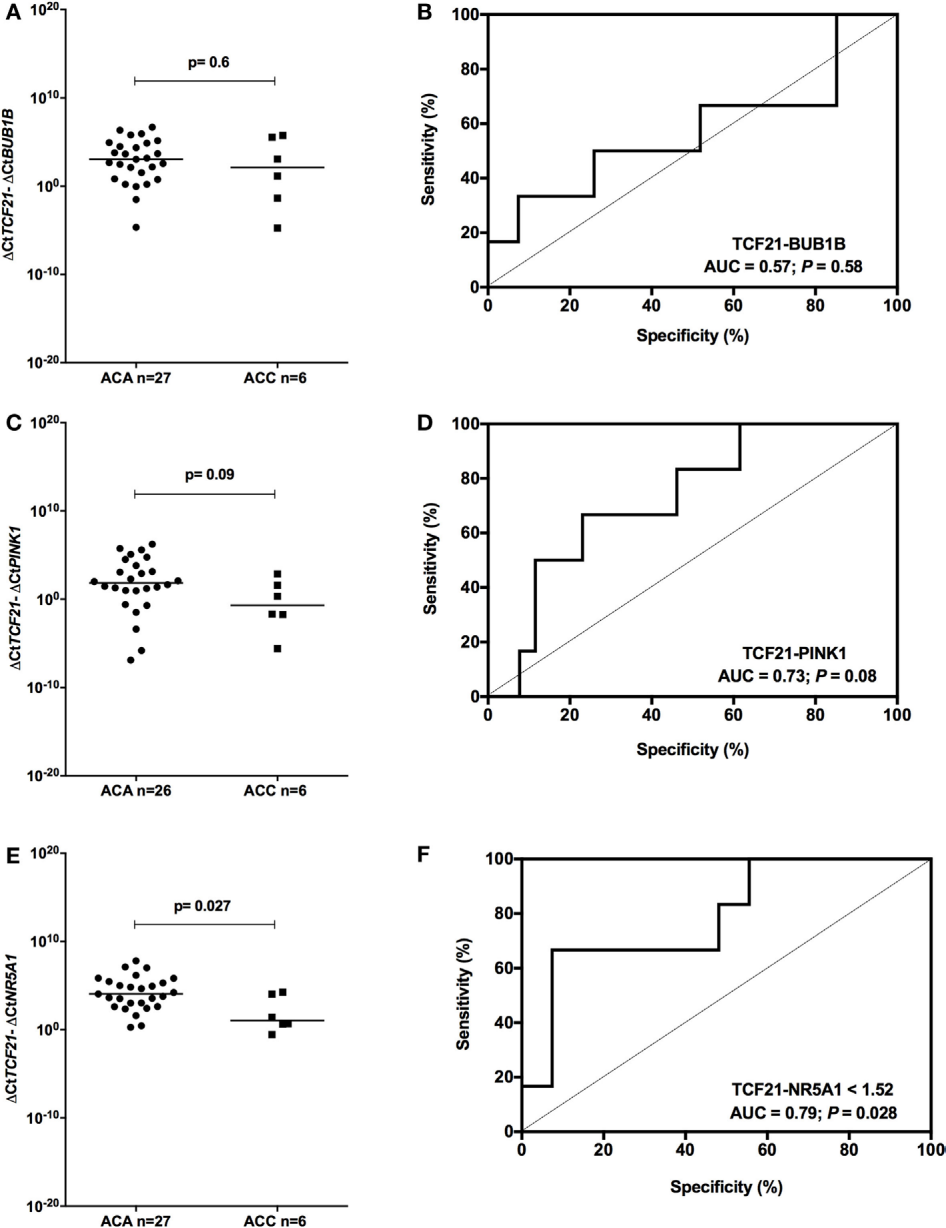
Analysis of the subtraction gene expression of pediatric patients aged <5 years with adenomas (ACAs) and carcinomas (ACCs) to determine the prognosis. **(A)** Δ*CtTCF21* and Δ*CtBUB1B* expression [median = −0.858; 95% confidence interval (CI): −4.799 to 2.314; *P* = 0.6] and **(B)** ROC curve test for *TCF21-BUB1B*; **(C)** Δ*CtTCF21* and Δ*CtPINK1* expression (median = −2.718; 95% CI: −5.263 to 0.577; *P* = 0.08) and **(D)** ROC curve test for *TCF21-PINK1*; **(E)** Δ*CtTCF21* and Δ*CtNR5A1* expression (median = −2.378; 95% CI: −4.323 to 0.588; *P* = 0.027) and **(F)** ROC curve test for *TCF21-NR5A1*.

To determine whether *TCF21* could be used as a molecular predictor of malignancy in pediatric patients, we analyzed the subtraction expression level of *TCF21-BUB1B, TCF21-PINK1*, and *TCF21-NR5A1* in the pediatric cohort. These analyses did not show significant differences in the tumors of patients aged >5 years (Figure 7). In the cohort of pediatric patients aged <5 years, the subtraction expression level of *TCF21-BUB1B* and *TCF21-PINK1* did not present significant differences (Figures 8A–D). However, *ΔCtTCF21* − *ΔCtNR5A1* (Figures 8E,F) showed significant differences (*P* = 0.026; Mann–Whitney test) between adenomas (median = 4.06; *n* = 27) and carcinomas (median = 1.06; *n* = 6). The AUC value was 0.79 (95% CI: 0.59 to 0.99; *P* = 0.028), and the cut-off determined was <1.52 with 66.7% of sensitivity and 92.6% of specificity. A poor outcome occurs when *ΔCtTCF21* − *ΔCtNR5A1* is <1.52. Due to the small number of carcinomas studied, in this case, the cutoff value was not applied as a predictor of OS.

### The OS for Patients Aged <5 Years Is Markedly Favorable

**Figure 9:**
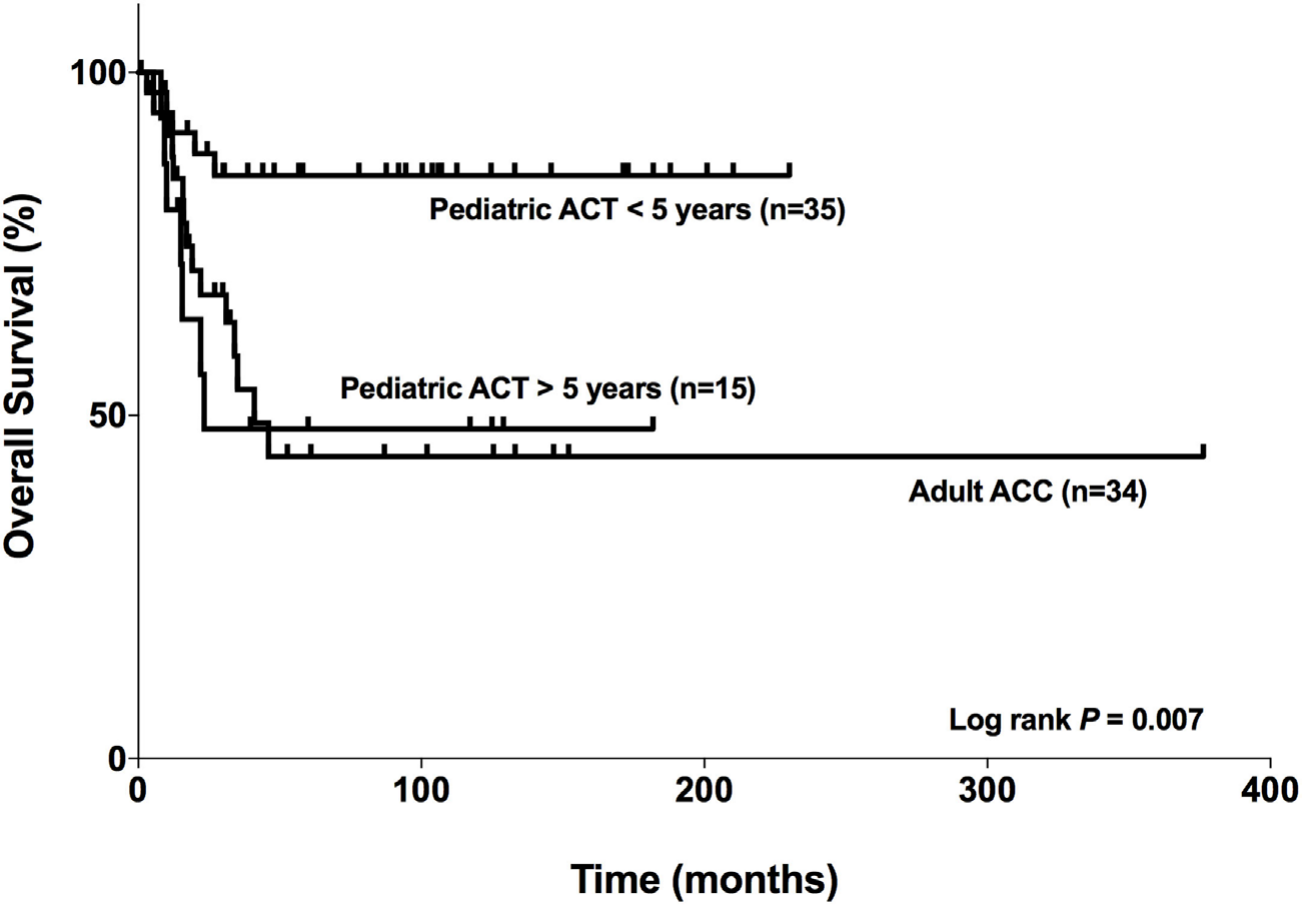
Comparison of the overall survival of adults with adrenocortical carcinomas (ACCs) and pediatric patients with adrenocortical tumors (ACTs). Adult patients with carcinoma (*n* = 34), pediatric patients aged <5 years with ACTs (*n* = 35), and pediatric patients aged >5 years with ACTs (*n* = 15).

In our cohort, the median survival for adult patients with carcinoma was 41 months for adults (Figure 9). For pediatric patients aged >5 years with ACTs, the median survival was 23.3 months, while the median survival for patients aged <5 years was 197.7 months. However, it was limited to the maximum follow-up time that was 230.07 months for this group. Therefore, as seen in Figure 9, the OS curves were significantly different (log rank *P* = 0.007) among the patients studied. In addition, the OS for patients aged <5 years was markedly favorable compared with that for patients >5 years and adult patients with carcinoma.

**Figure 10:**
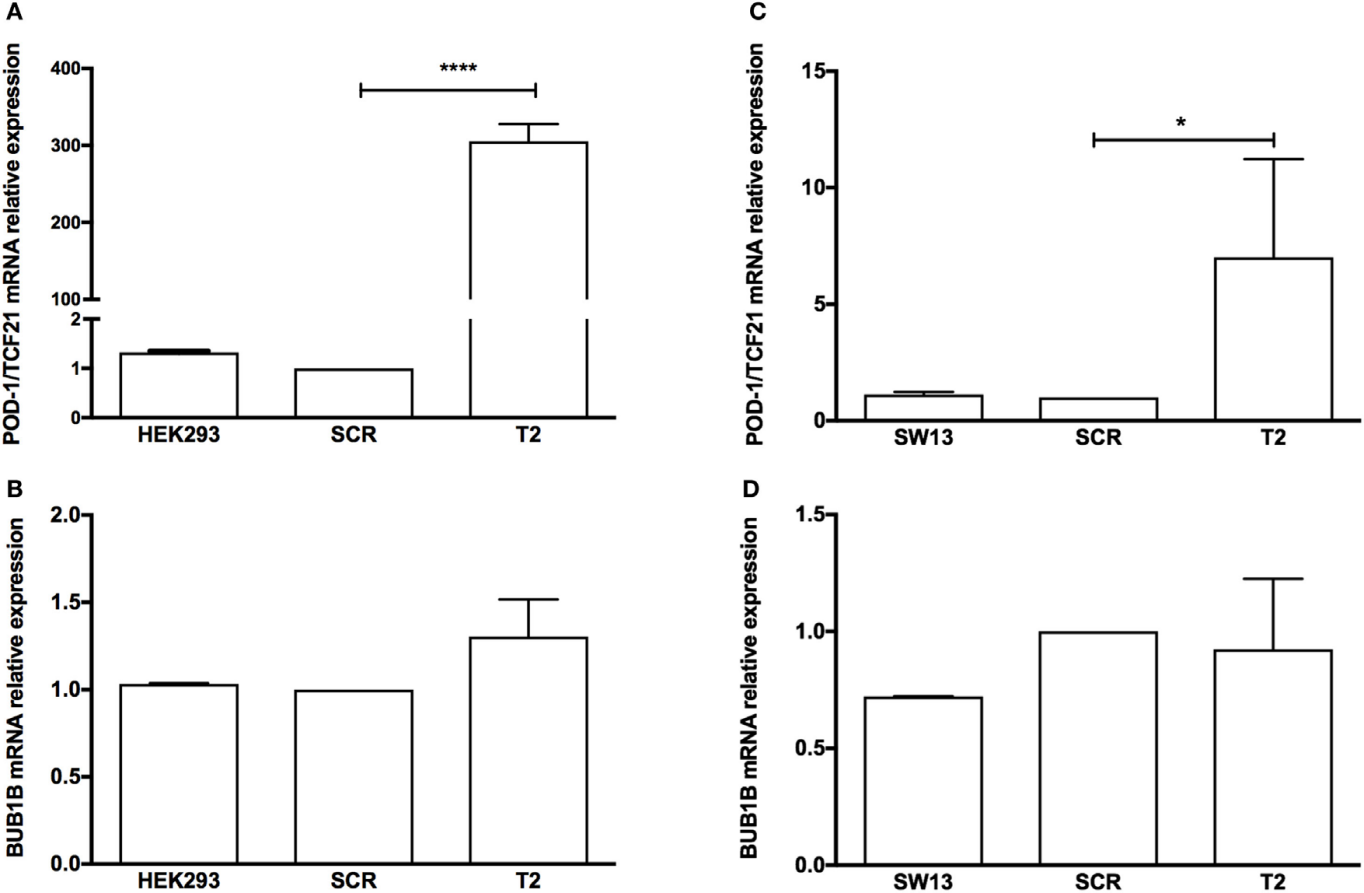
RT-qPCR analysis to determine the relative gene expression of **(A)** transcription factor 21 (TCF21), **(B)** BUB1B in HEK-293, HEK-SCR (scramble), and HEK-T2 cells (signal guide RNA-sgRNAT2), **(C)** TCF21, and **(D)** BUB1B in SW13, SW13-SCR, and SW13-T2 cells. Statistical significance was assessed by ANOVA test on three pairs.

## Discussion

Different authors have relied on RNA-based global gene expression profiles to identify molecular markers that differentiate malignant and benign adrenal tumors. Among these studies, de Reyniès et al. (14) identified three molecular markers in adult ACTs, *DLGAP5, BUB1B*, and *PINK1*, and found that *BUB1B* is overexpressed in carcinomas. According to their data, the combined expression of *BUB1B* and *PINK1* was the best predictor of OS among carcinomas. Thereafter, *BUB1B-PINK1* expression was validated in a different cohort of adult and pediatric patients (15). Giordano et al. (4) identified the downregulation of *TCF21* in ACCs in a microarray assay. In Franca et al. (10), we showed that *TCF21* was markedly downregulated in adult ACCs compared with adenomas and normal tissue.

Here, we found that *TCF21* was downregulated while *BUB1B* was upregulated in adult ACCs, in agreement with previous studies (10, 14). A previous study proposed that, among other cell cycle genes, *BUB1B* is negatively correlated with *TCF21* (10). We tested the causal relationship between *TCF21* and *BUB1B* and could not confirm the negative correlation between these genes in different experimental approaches and cell lines overexpressing *TCF21*.

We also analyzed whether the subtraction expression level of *TCF21-BUB1B* and *TCF21-PINK1* could distinguish between adenomas and carcinomas. The study of the subtraction expression levels of *BUB1B-PINK1* and *TCF21-BUB1B* discriminated between adult adenoma and carcinoma in a similar pattern. In addition, among adult malignant tumors, the combined expression of *TCF21* and *BUB1B* was a good predictor of OS. Accordingly, both *BUB1B-PINK1* and *TCF21-BUB1B* seem reliable molecular markers to be used in the clinical evaluation of adult adrenal tumors.

We employed these new molecular markers and the combined expression of *TCF21-NR5A1* to discriminate between benignant and malignant tumors in a cohort of pediatric ACTs. Increased *NR5A1* copy number has been associated with childhood adrenocortical tumorigenesis (31, 32), although this increase does not correlate with NR5A1 protein levels (33). The functional role of NR5A1 extends beyond steroidogenesis because NR5A1 regulates proliferation in adrenocortical cells, angiogenesis, extracellular matrix adhesion, cytoskeleton dynamics, and apoptosis in the adrenal cortex (34). Although there was no difference in the *NR5A1* expression of adenomas and carcinomas in the cohort of patients <5 years of age, the subtraction expression level of *TCF21-NR5A1* discriminates between benign and malignant tumors and may provide relevant information in addition to pathology analysis.

It is largely accepted that children have a better outcome than adolescents. As observed in the study by Wieneke and collaborators (18) in 83 pediatric tumors, there appears to be a biphasic age distribution with a poor clinical outcome in the group aged >5 years. In fact, we showed that the OS for patients aged <5 years was markedly favorable compared with that for patients aged >5 years and adult patients with carcinoma. This observation was utilized in the more recent study by Cecchetto et al. (35), where patients were separated in three different groups: <4 years, 4–10 years, and >10 years. Patients aged ≤4 years had a better outcome than the older ones. Indeed, <5 years patients have specific features distinguishing them from adolescent and adult tumors considering their genomic profiles and pathological and genetic mechanisms (36). Therefore, we suggest that separation between children and adolescents should be considered in future analysis.

In summary, we could not establish a consistent relationship between the analyzed genes for adult and pediatric tumors, although *TCF21* transfection in the H295R cell line has shown a tendency of reduction in *BUB1B* expression. In addition, the subtraction of gene expression of *TCF21 and BUB1B* can be a good predictor of OS in adult carcinomas, whereas the *TCF21-NR5A1* can be a molecular predictor of malignancy in pediatric ACTs. Moreover, we confirm that patients aged <5 years showed more favorable OS than adolescent patients. Finally, our study suggested a role of *TCF21* in ACTs that should be explored in future studies.

## Ethics Statement

This study was approved by the Ethics Committees of Hospital das Clinicas, Institute of Biomedical Sciences and Department of Pediatrics and School of Medicine of Ribeirão Preto, São Paulo, Brazil. Written informed consent was obtained from all the patients or from their parents.

## Author Contributions

BP performed the experiments, analyzed the data, and wrote the manuscript; SA provided the pediatric samples; MA provided the adult samples; MF provided the adult and pediatric samples; CL idealized the study, analyzed the data, and wrote the manuscript.

## Conflict of Interest Statement

The authors declare that there is no conflict of interest that could be perceived as prejudicing the impartiality of the research reported.

## Abbreviations

ACA, adrenocortical adenoma; ACC, adrenocortical carcinoma; ACT, adrenocortical tumor; ACTB, beta-actin; BUB1B, budding uninhibited by benzimidazoles 1 homolog beta; GFP, green fluorescent protein; GUSB, glucuronidase beta; NR5A1, nuclear receptor subfamily 5 group A member 1; PINK1, PTEN-induced putative kinase 1; POD1, podocyte-expressed 1; SF1, steroidogenic factor 1; TCF21, transcription factor 21.
