# Supplementary material for: TCF21/POD-1, a Transcritional Regulator of SF-1/NR5A1, as a Potential Prognosis Marker in Adult and Pediatric Adrenocortical Tumors
Source: Front Endocrinol (Lausanne). 2018 Feb 22;9:38. doi: 10.3389/fendo.2018.00038 (PMC5827685; doi:10.3389/fendo.2018.00038)
Supplement: Supplementary file 1 [file Table_1.docx]

| **Table S1 -** Clinical and molecular data of adult patients (n=78) | | | | | | | | | |
| --- | --- | --- | --- | --- | --- | --- | --- | --- | --- |
| **Patient** | **Age (Years)** | **Sex** | **Weiss**  **Score** | **Final**  **Diagnosis** | ***∆CtBUB1B-∆CtPINK1*** | ***∆CtTCF21-***  ***∆CtBUB1B*** | ***∆CtTCF21-***  ***∆CtPINK1*** | **CRD** | **Follow-up (months)** |
| B1 | 34.30 | F | 1 | ACA | 3.671 | -3.183 | 0.488 | 0 | 18.57 |
| B2 | 72.40 | M | 1 | ACA | 3.536 | -5.473 | -1.937 | 0 | 52.20 |
| B3 | 34.60 | M | 0 | ACA | 4.438 | -4.422 | 0.017 | 0 | 1.3 |
| B4 | 18.00 | F | 2 | ACA | 0.430 | -0.903 | -0.473 | 0 | 153.00 |
| B5 | 37.00 | F | 0 | ACA | 1.084 | -3.889 | -2.805 | 0 | 135.00 |
| B6 | 74.70 | F | 0 | ACA | 1.634* | -2.730 | -1.096 | 0 | 36.37 |
| B7 | 27.00 | F | 2 | ACA | 3.640 | -4.134 | -0.494 | 0 | 91.00 |
| B8 | 34.00 | F | 0 | ACA | 3.114 | -4.621 | -1.506 | 0 | 0.00 |
| B9 | 29.00 | F | 2 | ACA | 2.709 | -4.411 | -1.701 | 0 | 187.00 |
| B10 | 21.80 | F | 0 | ACA | 0.955 | 1.845 | 2.800 | 0 | 28.53 |
| B11 | 38.00 | F | 2 | ACA | 2.948 | -4.107 | -1.159 | 0 | 229.10 |
| B12 | 26.00 | F | 1 | ACA | 4.165 | -10.705 | -6.540 | 0 | 68.00 |
| B13 | 66.40 | F | 1 | ACA | 3.348 | -2.939 | 0.409 | 0 | 0.00 |
| B14 | 50.00 | F | 4 | ACA | 0.925* | 0.166 | 1.091 | 0 | 152.10 |
| B15 | 37.00 | F | 1 | ACA | 2.633 | -5.834 | -3.201 | 0 | 54.00 |
| B16 | 47.00 | F | 0 | ACA | ND | ND | ND | 0 | 98.23 |
| B17 | 48.10 | F | 1 | ACA | 3.277 | -5.699 | -2.422 | 0 | 121.00 |
| B18 | 40.00 | F | 0 | ACA | 2.002 | -6.293 | -4.291 | 0 | 211.83 |
| B19 | 23.90 | F | 2 | ACA | 1.353 | -4.764 | -3.411 | 0 | 40.97 |
| B20 | 41.00 | F | 0 | ACA | 4.572 | -2.081 | 2.491 | 0 | 79.03 |
| B21 | 64.00 | F | 0 | ACA | 3.740* | -2.837 | 0.902 | 0 | 46.00 |
| B22 | 31.60 | F | 2 | ACA | -0.136 | -1.588 | -1.724 | 0 | 0.00 |
| B23 | 49.60 | F | 0 | ACA | 4.071 | -2.424 | 1.647 | 0 | 4.43 |
| B24 | 27.00 | F | 0 | ACA | 4.258 | 0.175 | 4.433 | 0 | 1.00 |
| B25 | 37.00 | F | 1 | ACA | 2.224 | -0.617 | 1.607 | 0 | 127.00 |
| B26 | 39.00 | F | 1 | ACA | ND | ND | ND | 0 | 93.00 |
| B27 | 58.80 | F | 0 | ACA | 3.808 | -2.833 | 0.975 | 0 | 6.80 |
| B28 | 36.00 | F | 0 | ACA | 0.522 | -2.691 | -2.169 | 0 | 45.17 |
| B29 | 56 | M | 0 | ACA | 1.593 | -3.877 | -2.283 | ND | 22.1 |
| B30 | 39 | F | 0 | ACA | 0.925* | 0.190 | 1.115 | ND | 33.3 |
| B31 | 54 | M | 0 | ACA | 4.989 | -5.744 | -0.755 | 1 | 0.0 |
| B32 | 46 | F | 1 | ACA | 2.451 | -3.105 | -0.654 | ND | 29.7 |
| B33 | 59 | M | 0 | ACA | 1.944 | -3.726 | -1.783 | ND | 24.5 |
| B34 | 48 | M | 2 | ACA | 1.351* | -4.361 | -3.010 | ND | 23.5 |
| B35 | 34 | M | 0 | ACA | 1.396 | -2.047 | -0.651 | ND | 19.7 |
| B36 | 27 | F | 0 | ACA | 9.167* | -3.446 | 5.720 | ND | 18.3 |
| B37 | 30 | F | 0 | ACA | 12.089 | -9.625 | 2.465 | ND | 11.8 |
| B38 | 43 | M | 1 | ACA | 2.230 | -3.046 | -0.815 | ND | *38.9* |
| B39 | 31 | F | ND | ACA | 2.272 | -1.767 | 0.505 | ND | 29.9 |
| B40 | 56 | F | 0 | ACA | 1.534 | -3.368 | -1.834 | ND | 25.4 |
| B41 | 31 | F | 1 | ACA | 9.008* | -7.966 | 1.042 | 0 | 10.1 |
| B42 | 24.00 | F | 2 | ACA | 0.709 | -1.646 | -0.937 | 0 | 195.87 |
| B43 | 21.50 | F | 0 | ACA | 2.012* | -3.932 | -1.920 | 0 | 24.90 |
| B44 | 43.20 | F | 0 | ACA | 5.094 | -3.491 | 1.603 | 0 | 24.60 |
| B45 | 32.00 | F | 8 | ACC | -3.182 | 6.919 | 3.737 | 1 | 12.40 |
| B46 | 22.40 | F | 6 | ACC | 0.056 | 3.397 | 3.453 | 0 | 29.83 |
| B47 | 66.50 | M | 8 | ACC | -4.013 | -3.099 | -7.111 | 0 | 13.6 |
| B48 | 62.80 | F | 7 | ACC | -1.888* | 1.521 | -0.367 | 1 | 15.80 |
| B49 | 29.00 | F | 3 | ACC | 1.052 | -0.992 | 0.061 | 0 | 61.00 |
| B50 | 31.00 | F | 7 | ACC | -2.541* | 4.270 | 1.730 | 1 | 16.00 |
| B51 | 22.70 | F | 8 | ACC | -6.102 | 9.202 | 3.101 | 1 | 22.00 |
| B52 | 33.00 | M | 5 | ACC | -0.176 | 3.742 | 3.567 | 0 | 16.00 |
| B53 | 35.00 | F | 3 | ACC | 2.858 | -4.120 | -1.262 | 0 | 133.17 |
| B54 | 30.00 | M | 6 | ACC | -1.261 | 6.438 | 5.176 | 1 | 12.00 |
| B55 | 55.50 | F | 8 | ACC | -3.761 | 1.880 | -1.881 | 1 | 35.10 |
| B56 | 19.00 | F | 6 | ACC | -4.107 | 5.467 | 1.359 | 0 | 102.00 |
| B57 | 45.00 | F | 8 | ACC | -3.104 | 1.193 | -1.911 | 0 | 152.00 |
| B58 | 23.10 | F | 5 | ACC | 1.200* | 3.024 | 4.224 | 0 | 52.70 |
| B59 | 19.00 | F | 4 | ACC | 3.495 | -4.492 | -0.997 | 0 | 125.50 |
| B60 | 23.00 | F | 6 | ACC | -3.468 | 4.837 | 1.369 | 1 | 17.00 |
| B61 | 42.80 | F | 3 | ACC | 1.450 | 3.678 | 5.129 | 0 | 17.93 |
| B62 | 29.00 | F | 6 | ACC | ND | ND | ND | 1 | 31.00 |
| B63 | 37.70 | F | 5 | ACC | -7.530 | 0.194 | -7.336 | 1 | 34.00 |
| B64 | 66.00 | F | 4 | ACC | 1.299 | -3.032 | -1.733 | 0 | 86.97 |
| B65 | 40.00 | F | 4 | ACC | 0.452* | -2.019 | -1.566 | 0 | 376.23 |
| B66 | 37.40 | F | 4 | ACC | 2.139 | -3.770 | -1.631 | 0 | 27.00 |
| B67 | 53.90 | M | 3 | ACC | 4.174 | 0.192 | 4.366 | 0 | 32.20 |
| B68 | 69 | F | 3 | ACC | 10.303 | -9.080 | 1.222 | 0 | 31.0 |
| B69 | 39 | M | 8 | ACC | 1.524 | 0.702 | 2.227 | 1 | 19.0 |
| B70 | 83 | M | 8 | ACC | -2.879* | 1.294 | -1.585 | 1 | 3.0 |
| B71 | 33 | F | 6 | ACC | -0.426 | 3.046 | 2.620 | 1 | 41.0 |
| B72 | 47 | M | 7 | ACC | 2.339 | 3.231 | 5.570 | 0 | 146.7 |
| B73 | 54 | M | 3 | ACC | 6.273 | -4.723 | 1.550 | 0 | 9.5 |
| B74 | 53 | M | 9 | ACC | -1.621* | 0.460 | -1.161 | 1 | 10.4 |
| B75 | 66 | M | 6 | ACC | -3.213 | 5.933 | 2.720 | 0 | 4.5 |
| B76 | 22.00 | F | 7 | ACC | -2.489 | 6.078 | 3.588 | 1 | 10.00 |
| B77 | 44.00 | F | 8 | ACC | -2.665 | 1.699 | -0.966 | 1 | 46.00 |
| B78 | 51.20 | M | 3 | ACC | 3.082 | -2.208 | 0.874 | 0 | 32.43 |

**ND – no data; CRD – cancer-related death (0 – without; 1- with); *data excluded due overlap**
