# Supplementary material for: TCF21/POD-1, a Transcritional Regulator of SF-1/NR5A1, as a Potential Prognosis Marker in Adult and Pediatric Adrenocortical Tumors
Source: Front Endocrinol (Lausanne). 2018 Feb 22;9:38. doi: 10.3389/fendo.2018.00038 (PMC5827685; doi:10.3389/fendo.2018.00038)
Supplement: Supplementary file 2 [file Table_2.docx]

| **Table S2 -** Clinical and molecular data of pediatric patients | | | | | | | | | |
| --- | --- | --- | --- | --- | --- | --- | --- | --- | --- |
|  | Patients < 5 years (n= 35) | | | | | | | | |
| **Patient** | **Age**  **(Years)** | **Sex** | **Weiss**  **Score** | **Final**  **Diagnosis** | ***∆CtTCF21-***  ***∆CtBUB1B*** | ***∆CtTCF21-***  ***∆CtNR5A1*** | ***∆CtTCF21-***  ***∆CtPINK1*** | **CRD** | **Follow-up**  **(months)** |
| Ped1 | 2.78 | F | 0 | ACA | 3.791 | 5.834 | 5.598 | 0 | 56.73 |
| Ped2 | 0.83 | M | 1 | ACA | 3.651 | 3.780 | 2.087 | 0 | 94.53 |
| Ped3 | 3.33 | F | 4 | ACA | 1.537 | 4.658 | 3.817 | 0 | 87.63 |
| Ped4 | 1.97 | F | 4 | ACA | 0.829 | 3.638 | 2.288 | 0 | 38.7 |
| Ped5 | 1.23 | F | 8 | ACA | 5.938 | 7.820 | 4.767 | 0 | 24.36 |
| Ped6 | 1.97 | F | 4 | ACA | 2.169 | 7.014 | 6.241 | 0 | 103.86 |
| Ped7 | 0.43 | F | 8 | ACA | 3.676 | 5.301 | 3.146 | 0 | 100.33 |
| Ped8 | 2.93 | M | 6 | ACA | 2.476 | 4.841 | 3.071 | 0 | 145.83 |
| Ped9 | 2.56 | M | 7 | ACA | 2.126 | 2.431 | ND | 0 | 230.07 |
| Ped10 | 0.44 | F | 4 | ACA | 4.941 | 6.169 | 5.097 | 0 | 210.23 |
| Ped11 | 1.44 | F | 7 | ACA | 6.344 | 7.125 | 5.755 | 0 | 58.13 |
| Ped12 | 1.30 | M | 2 | ACA | 0.214 | 5.858 | 0.990 | 0 | 173.00 |
| Ped13 | 2.30 | F | 5 | ACA | 0.760 | 0.285 | -5.796 | 0 | 107.17 |
| Ped14 | 0.90 | F | 3 | ACA | 2.571 | 3.031 | 1.373 | 0 | 133.00 |
| Ped15 | 2.10 | F | 1 | ACA | 3.043 | 5.012 | -1.479 | 0 | 92.00 |
| Ped16 | 2.20 | M | 6 | ACA | 0.218 | 1.595 | -0.696 | 0 | 17.40 |
| Ped17 | 2.20 | M | 4 | ACA | 5.798 | 2.611 | 4.506 | 0 | 188.00 |
| Ped18 | 2.10 | M | 5 | ACA | 4.498 | 2.366 | 1.656 | 0 | 107.00 |
| Ped19 | 1.30 | M | 7 | ACA | 4.875 | 4.224 | 2.915 | 0 | 171.30 |
| Ped20 | 2.50 | F | 6 | ACA | 6.678 | 3.531 | 1.213 | 0 | 124.70 |
| Ped21 | 1.60 | F | ND | ACA | 5.153 | 4.930 | 2.003 | 0 | 1.00 |
| Ped22 | 1.60 | F | 3 | ACA | -1.516 | 2.633 | -3.374 | 0 | 43.97 |
| Ped23 | 1.20 | F | 5 | ACA | -0.046 | 3.037 | -0.594 | 0 | 30.00 |
| Ped24 | 1.00 | F | 6 | ACA | 4.368 | 3.571 | 1.462 | 0 | 30.40 |
| Ped25 | 2.80 | F | 3 | ACA | 3.180 | 4.057 | 0.962 | 0 | 182.00 |
| Ped26 | 2.20 | F | 2 | ACA | 2.674 | 5.465 | 1.302 | 0 | 48.00 |
| Ped27 | 2.50 | F | 1 | ACA | -4.658 | 0.452 | -6.882 | 0 | 105.97 |
| Ped28 | 3.83 | M | 6 | ACC | ND | ND | ND | 0 | 112.5 |
| Ped29 | 2.93 | M | 7 | ACC | ND | ND | ND | 1 | 5.5 |
| Ped30 | 1.54 | F | 7 | ACC | 1.140 | 4.035 | 2.866 | 1 | 5.4 |
| Ped31 | 2.60 | M | 7 | ACC | -1.371 | 0.656 | -5.575 | 1 | 12.00 |
| Ped32 | 0.90 | F | 4 | ACC | 5.753 | 4.254 | 1.589 | 0 | 78.00 |
| Ped33 | 2.00 | F | 8 | ACC | 3.076 | 0.688 | -1.689 | 1 | 27.00 |
| Ped34 | 3.10 | F | 7 | ACC | 5.552 | 1.437 | 0.334 | 1 | 20.00 |
| Ped35 | 2.60 | M | 5 | ACC | -4.752 | -0.558 | -1.742 | 0 | 200.97 |
|  | Pediatric >5 years n= 15 | | | | | | | | |
| Ped36 | 7.09 | F | 1 | ACA | -1.619 | 0.504 | -0.682 | 0 | 181.90 |
| Ped37 | 16.90 | F | 2 | ACA | -0.077 | 3.963 | 3.278 | 0 | 39.60 |
| Ped38 | 13.00 | F | 1 | ACA | 1.959 | 4.275 | 4.411 | 0 | 14.80 |
| Ped39 | 9.00 | M | 2 | ACA | 0.610 | 2.341 | 4.693 | 0 | 117.20 |
| Ped40 | 6.00 | F | 2 | ACA | 0.011 | 1.973 | -3.678 | 0 | 129.00 |
| Ped41 | 5.54 | F | 7 | ACC | 4.967 | 6.246 | 6.853 | 1 | 15.50 |
| Ped42 | 15.56 | M | 7 | ACC | 3.850 | 5.034 | 2.767 | 1 | 9.27 |
| Ped43 | 9.90 | F | 9 | ACC | 5.170 | 6.780 | 5.292 | 0 | 14.00 |
| Ped44 | 8.39 | F | 6 | ACC | ND | ND | ND | 1 | 23.27 |
| Ped45 | 12.31 | F | 4 | ACC | -1.362 | 3.596 | 3.805 | 1 | 15.03 |
| Ped46 | 16.02 | F | 7 | ACC | -1.328 | 0.550 | -1.763 | 1 | 10.00 |
| Ped47 | 17.00 | M | 7 | ACC | 2.657 | 4.271 | 4.717 | 0 | 60.03 |
| Ped48 | 15.00 | F | 8 | ACC | 5.876 | 3.623 | 0.997 | 1 | 22.00 |
| Ped49 | 17.70 | F | 8 | ACC | ND | 5.208 | ND | 1 | 8.00 |
| Ped50 | 17.00 | F | 4 | ACC | 0.828 | 3.187 | 3.093 | 0 | 125.00 |

**ND – no data; CRD – cancer-related death (0 – without; 1- with)**
